# Intraoperative dexamethasone and delirium after cardiac surgery: a randomized clinical trial

**DOI:** 10.1186/cc12334

**Published:** 2013-03-19

**Authors:** AC Sauër, AJ Slooter, DS Veldhuijzen, MM Van Eijk, D Van Dijk

**Affiliations:** 1 University Medical Center Utrecht, the Netherlands

## Introduction

The objective of this study is to investigate the effect of intraoperative administration of dexamethasone versus placebo on the incidence of delirium in the first four postoperative days after cardiac surgery.

## Methods

Within the context of the large multicenter Dexamethasone for Cardiac Surgery (DECS) trial [1] for which patients were randomized to 1 mg/kg dexamethasone or placebo at induction of anesthesia, a monocenter substudy was conducted. The primary outcome of this study was the incidence of delirium in the first four postoperative days. Secondary outcomes were duration of delirium, use of restrictive measures and sedative, antipsychotic and analgesic requirements. Delirium was assessed daily by trained research personnel, using the Richmond Agitation Sedation Scale and the Confusion Assessment Method. Medical, nursing and medication charts were evaluated for signs of delirium and use of prespecified medication. Analysis was by intention to treat.

## Results

Of 768 eligible patients, complete data on delirium could be collected in 738 patients. The incidence of delirium was 14.2% in the dexamethasone group and 14.9% in the placebo group (odds ratio = 0.95, 95% CI = 0.63 to 1.43). No significant difference was found on the duration of delirium between the intervention (median = 2 days, interquartile range 1 to 3 days) and placebo (median = 2 days, interquartile range 1 to 2 days) group (*P *= 0.45). The use of restrictive measures and administration of sedatives, haloperidol, benzodiazepine and opiates were comparable between both groups.

## Conclusion

Intraoperative injection of dexamethasone seems not to affect the incidence or duration of delirium in the first 4 days after cardiac surgery, suggesting this regimen is safe to use in the operative setting with respect to psychiatric adverse events.
